# Effects of forest age and stand density on the growth, soil moisture content, and soil carbon content of *Populus simoni* plantations in the sandy area of western Liaoning, Northeast China

**DOI:** 10.1038/s41598-025-86215-4

**Published:** 2025-01-20

**Authors:** Hao Chen, Zhaowei Zhang, Xiangyu Yang, Xin Ai, Yutao Wang, Ping Liu

**Affiliations:** 1 College of Forestry, Shenyang Agricultural University, Shenyang, 110866 China; 2 Key Laboratory for Silviculture of Liaoning Province, Shenyang Agricultural University, Shenyang, 110866 China

**Keywords:** Western Liaoning sandy land, Stand growth, Forest management, Soil carbon stock, Ecology, Plant sciences, Ecology

## Abstract

Poplar (*Populus simoni*) plantations are crucial in the sandy regions of western Liaoning, serving key roles in wind protection, sand stabilization, soil moisture regulation, and carbon sequestration. However, challenges such as suboptimal stand quality and limited ecological benefits persist. This study aims to elucidate the growth dynamics of poplar plantations and their impact on soil moisture content and soil carbon content in this region. We established 75 standard plots across various age groups and stand densities in Fuxin City, measuring poplar diameter at breast height (DBH), tree height (TH), soil moisture content, and soil carbon content. We found that DBH and TH increase with increasing stand density in young and middle-aged forests, but the opposite is true at near-maturity, maturity, and over-maturity, where DBH and TH decrease with increasing stand density. Soil moisture content rises with stand density in younger forests, while soil carbon content increases with age, with surface soil layers exhibiting higher carbon concentrations. The soil carbon stock in these plantations is approximately 3.0 × 10^6^ tons, the highest recorded in Fuxin City. This research provides a foundation for the effective management and development of poplar plantations in wind-prone, sandy areas. Overall, optimizing stand density and managing forest age distribution are essential for enhancing the ecological and carbon sequestration benefits of poplar plantations in this region.

## Introduction

Global warming and increasing weather extremes have exacerbated tree mortality and forest degradation^1^, posing significant challenges to forest ecosystems, particularly as drought conditions intensify^2^. The western region of Liaoning Province, characterized by its sandy terrain, is ecologically vulnerable. This region encompasses approximately 390,000 hectares of sandy land and 490,000 hectares of desertified land, with annual soil erosion losses reaching 131 million tons^3^. In response to these challenges, China initiated the Three-North Protective Forest System in 1979 to combat desertification and sandstorms in the northeastern, northwestern, and northern regions^4,5^. Additionally, since 2022, Liaoning Province has embarked on an ambitious plan to expand forest cover by 1.41 million mu and enhance forest areas by 85,000 hectares over three years. The Three-North Protective Forest System has been instrumental in mitigating wind and sand, improving land conditions, safeguarding agriculture and livestock, and fostering regional ecological, economic, and social development^6^. However, insufficient rainfall and high evapotranspiration rates in these regions continue to impede successful afforestation, negatively impacting survival rates and hindering the high-quality growth of forest stands, thereby reducing their carbon sequestration potential^7,8^. Poplar (*Populus simoni*) are a primary species utilized in creating protective forests in western Liaoning, with a planting area of approximately 24,000 hectares. These plantations play a critical role in wind and sand prevention, soil and water conservation, microclimate improvement, and carbon and oxygen cycling. In light of growing concerns about climate change and carbon emissions, China has adopted a dual-carbon strategy in forestry, aiming to respond proactively to climate change and achieve carbon peaking and carbon neutrality. This strategy emphasizes the protection and expansion of forest resources, enhancement of carbon sinks, and promotion of low-carbon development. Studies indicate that a 20 cm diameter poplar tree can sequester 172 kg of carbon dioxide, release 125 kg of oxygen, and capture 16 kg of dust annually^9^. This underscores the significant carbon sink function and growth potential of poplar trees within the dual-carbon strategy framework. Understanding how different stand densities affect poplar tree growth across various age groups is crucial. This knowledge will assist in selecting optimal stand densities for silvicultural practices, enhancing tree growth rates and quality, and managing stand density to minimize competition, thereby improving resource acquisition and overall yield. Additionally, it will help optimize poplar’s role in controlling soil and water erosion and evaluating the impact of poplar plantations on carbon cycling. This understanding will provide valuable data for addressing climate change and optimizing the benefits of poplar forests.

Afforestation is widely acknowledged as a key strategy for enhancing terrestrial carbon sequestration and combating global warming^10^. New forests are crucial for offsetting the carbon sink impacts of forest degradation^11^. In warming and arid climates, forests play a vital role in the water and carbon cycles^12^. Soil moisture content is a critical indicator of forest ecosystem health, directly influencing forest growth, distribution, hydrological cycles, soil erosion, biodiversity, and soil organic carbon accumulation through its effects on plant productivity and decomposition rates^13^. Carbon stock refers to the amount of carbon stored in an ecosystem, including soil, vegetation, and the atmosphere^14^. Forestry significantly impacts the global carbon cycle and climate change. Recent research has focused on forest growth and carbon dynamics, including forest carbon reserves, stock changes, and density^15^, though less attention has been given to soil carbon content. Soil carbon pools, which represent the largest terrestrial carbon reservoirs, are highly sensitive to climate and land-use changes, management practices, and other factors. They play a crucial role in climate change mitigation through carbon sequestration^16^. Understanding the interplay between terrestrial ecosystems and global changes, including how land use and management affect soil organic carbon, is essential for recognizing the potential of agroecology and forest ecosystems^17,18^. Studies indicate that land use and management changes impact soil organic carbon 7–10 times more than climate change alone. Soil carbon content at various depths is crucial for assessing long-term impacts of forestry practices, and effective management is vital for maintaining soil health and quality^19–21^.

Poplar (*Populus simoni*), a key afforestation species in northern China, plays a vital role in stabilizing sandy soils, preventing erosion, and supporting ecological security in western Liaoning. Its deep and expansive root system makes it especially valuable in wind-prone areas, and it thrives in well-drained, sandy soils common to the region. Additionally, it contributes to national timber security and local economic development^22^. However, existing research on poplar plantations often focuses on specific age groups, with limited studies addressing varying stand densities throughout the full life cycle, particularly in wind-prone areas. This study aims to investigate the effects of stand density on diameter at DBH, TH, soil moisture content, and soil carbon content across juvenile, middle-aged, and mature poplar plantations. We hypothesize that stand density will enhance these factors in early stages but decrease them in mature forests, and that soil carbon content will increase with age while soil moisture content will decrease. By identifying optimal stand densities and improving management strategies, this research seeks to enhance the sustainability and efficiency of poplar planting, supporting ecological protection and sustainable land use in the sandy areas of western Liaoning.

## Materials and methods

### Study area

**Fig. 1:**
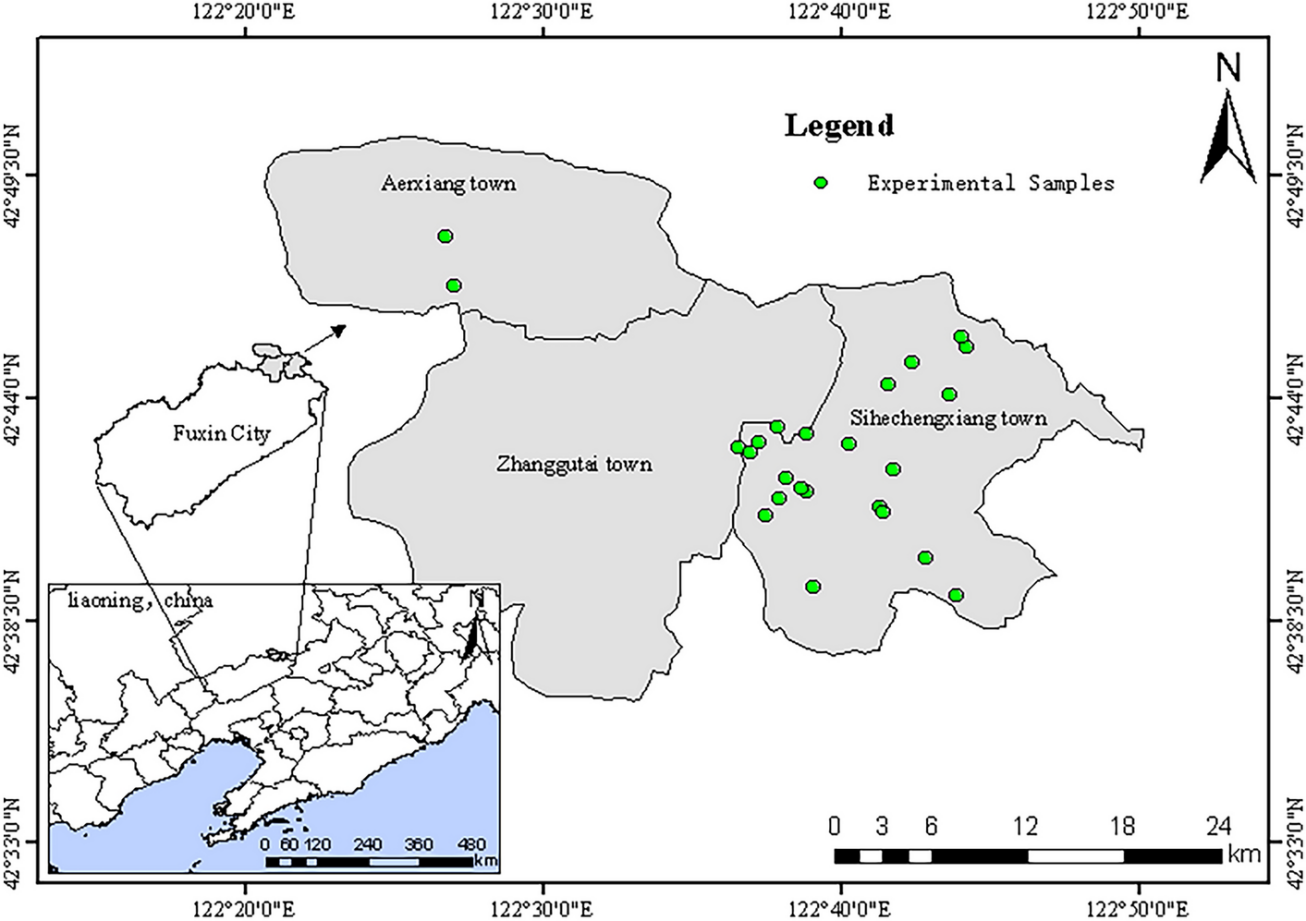
Experimental samples information.

The study was conducted in Zhangwu County, Fuxin City, Liaoning Province, a key area for the “Three Norths” protective forest project and a pilot county for its afforestation management. The region has a temperate monsoon continental climate, with large seasonal changes, rain and heat at the same time, plenty of sunshine, an average annual temperature of about 8.0 ℃, an average annual precipitation of 497.4 mm, windy and windy in the spring, with a long period of cold, and a large temperature difference between day and night. Situated on the southern edge of the Horqin Sandland, the study area is geographically located between 121°53′ and 122°58′ east longitude and 42°07′ and 42°51′ north latitude. The predominant soil types in the area include meadow soil, wind-blown sandy soil, brown loam, and brown soil. These soils generally develop from parent materials such as wind-deposited sands and residual materials from local bedrock. The soil’s texture ranges from sandy to loamy, with varying levels of organic matter and nutrients. The region’s sandy soils, in particular, are highly prone to wind erosion and nutrient leaching, requiring proper forest management and afforestation practices to improve soil stability and fertility. Representative vegetation consists of meadow grasses, while the main tree species are poplar (*Populus simoni*), willow (*Salix babylonica*), oil pine (*Pinus tabuliformis*), and camphor pine (*Pinus sylvestris* var. *mongolica*). The experimental sample map is shown in Fig. 1.

### Data collection

#### Plot survey

A forest survey was conducted in 2023 in the semi-arid sandy area of Zhangwu County, Fuxin City, Liaoning Province, to identify poplar plantations with representative growth conditions and environmental characteristics for experimental sampling. The study categorized poplar plantations into five age groups: young forests (1–10 years; YF), middle-aged forests (11–20 years; MAF), near-mature forests (21–25 years; NMF), mature forests (26–32 years; MF), and over-mature forests (greater than 32 years; OMF). Five sample plots were selected for each age group according to different stand densities: about 400 plants per hectare, 600 plants per hectare, 800 plants per hectare, 1000 plants per hectare and 1200 plants per hectare, for a total of 25 plots.

For each sample plot, basic information such as slope, slope direction, and soil type was recorded. An inclinometer was used to measure the slope angle, and a compass was employed to record the slope direction. Within each sample plot, three standard plots (20 m × 20 m each) were established as replicates for detailed investigation and soil sampling, resulting in a total of 75 standard plots. Measurement instruments, including a diameter at DBH ruler and a Bruleys altimeter, were used to assess tree diameter and height. Soil conditions were also investigated in each sample plot. Detailed information about the sample plots is presented in Table 1.

**Table 1:** Sample information.

Age	Stand density/(plants per hectare)	Number of plots	Average DBH/cm	Average height/(m)	Canopy density	Altitude/m	Slope/(°)	Slope position	Slope direction
YF	383	3	10.2 ± 2.2	9.5 ± 2	0.3	189	4	Level ground	No slope
YF	574	3	11.3 ± 2.4	10.2 ± 1.9	0.3	213	4	Level ground	No slope
YF	804	3	14 ± 2.8	11 ± 1.7	0.3	196	4	Level ground	No slope
YF	1008	3	13.2 ± 2.7	11.3 ± 2.2	0.3	177	4	Level ground	No slope
YF	1152	3	14.3 ± 2.4	11.6 ± 1.8	0.3	201	4	Level ground	No slope
MAF	414	3	14.6 ± 2.9	13 ± 2.1	0.4	159	4	Level ground	No slope
MAF	597	3	14.8 ± 2.5	14.3 ± 2.5	0.3	194	5	Level ground	No slope
MAF	800	3	16 ± 2.6	16.7 ± 1.7	0.4	146	4	Level ground	No slope
MAF	993	3	17.4 ± 2.5	17.5 ± 1.8	0.5	186	4	Level ground	No slope
MAF	1206	3	18 ± 2.3	15.7 ± 2.7	0.7	197	4	Level ground	No slope
NMF	406	3	22.3 ± 4.5	19 ± 3.5	0.6	190	4	Level ground	No slope
NMF	625	3	21.4 ± 3.6	18.7 ± 3	0.4	209	4	Level ground	No slope
NMF	773	3	20.2 ± 3.8	18.3 ± 2.6	0.5	175	4	Level ground	No slope
NMF	961	3	19.8 ± 3.2	18.1 ± 3.5	0.5	200	4	Level ground	No slope
NMF	1203	3	19.2 ± 3.8	18.4 ± 3.1	0.8	182	4	Level ground	No slope
MF	448	3	23.6 ± 3.6	20.7 ± 2.4	0.3	196	4	Level ground	No slope
MF	605	3	23.4 ± 3.7	19.2 ± 2.6	0.3	248	0	Level ground	No slope
MF	823	3	23.7 ± 4.5	18.3 ± 1.8	0.4	254	0	Level ground	No slope
MF	994	3	23.8 ± 4.5	19.8 ± 2.4	0.6	210	4	Level ground	No slope
MF	1189	3	22 ± 4.2	18.7 ± 2.7	0.4	252	0	Level ground	No slope
OMF	406	3	26.1 ± 5.3	20.4 ± 3.6	0.4	175	4	Level ground	No slope
OMF	578	3	18.9 ± 5.4	15.8 ± 4.1	0.7	199	4	Level ground	No slope
OMF	775	3	16.2 ± 3.7	13.8 ± 3.3	0.4	175	4	Level ground	No slope
OMF	1021	3	20.8 ± 6.1	13.1 ± 3.9	0.8	207	4	Level ground	No slope
OMF	1178	3	14.5 ± 3.7	15.8 ± 4.7	0.3	207	4	Level ground	No slope

DBH and height are mean ± sd.

#### Determination of soil moisture content

For each forest stand, a basic information survey was conducted, and a soil sampling point was established at the center of the understory vegetation layer within each standard plot. The surface debris was cleared, and a soil profile measuring 0.6 m in length, width, and depth was excavated. Soil samples were collected at fixed depths of 0–20 cm, 20–40 cm, and 40–60 cm using a 100 cm^3^ ring knife, resulting in a total of 225 soil samples. These samples were immediately weighed, placed in plastic bags for preservation, and transported to the laboratory.

In the laboratory, soil moisture content was determined using the drying and weighing method^23^. Soil samples were dried in an oven at 105 °C until they reached a constant weight. After cooling, the samples were weighed to measure soil moisture content. The soil moisture content was calculated using the following formula:

1 $$\begin{array}{c}SMC=\frac{\left({SMC}_{0}-{SMC}_{1}\right)}{{SMC}_{1}}\times 100\end{array}$$

In the formula, *SMC* represents the soil moisture content (%), *SMC*_*0*_ is the weight of the soil sample before drying (g), and *SMC*_*1*_ is the weight of the soil sample after drying (g).

#### Determination of soil carbon content

The organic carbon content of the soil samples was determined using the potassium dichromate-sulfuric acid oxidation method^24^. All measurements were performed in triplicate to ensure accuracy. The soil samples were ground and passed through a 100-mesh sieve. Soil from the same forest stand was sorted according to depths of 0–20 cm, 20–40 cm, and 40–60 cm, then mixed and bagged for analysis.

For the determination of soil organic carbon content, a 0.3 g sample was weighed using an electronic balance. To this sample, 5 ml of 1 mol L⁻^1^ potassium dichromate (K₂Cr₂O₇) solution was added, followed by 10 ml of concentrated sulfuric acid. After cooling, 50 ml of distilled water was added, along with 3 drops of o-phenanthroline indicator. The mixture was then titrated with 0.3 mol L⁻^1^ ferrous sulfate (FeSO₄) solution until a color change was observed. Six blank controls were prepared, and their average value was used for calibration.

The organic carbon content of the samples was calculated using the following formula:

2 $$\begin{array}{c}SOC=\frac{0.3\times \left({v}_{0}-v\right)\times 0.003\times 1.33}{0.025}\times 1000\end{array}$$

In the formula, *SOC* represents the soil organic carbon content (g kg^-1^), *v*_*0*_ is the volume of Fe_2_SO₄ standard solution used in the blank test (ml), and *v* is the volume of Fe_2_SO₄ standard solution used in the sample determination (ml).

#### Estimation of soil carbon stocks in poplar plantations in the sandy region of western Liaoning

Soil bulk weight was determined by the ring knife method and soil carbon stocks were calculated according to Eq. (3)^25^:

3 $${S}_{C}={\sum }_{i=0}^{n}\left(0.1{X}_{i}{L}_{i}{B}_{i}\right)$$

In the formula,* S*_*c*_ represents the soil carbon stock for the 0–60 cm soil layer (t ha^-1^);* i* is the soil layer sequence number; *X* is the soil carbon content (g kg^-1^); *L* is the soil layer thickness (cm); *B* is the soil bulk density (g cm^-3^); and 0.1 is a unit conversion factor.

According to the forest resources inventory data for Liaoning Province, there are 7,345 sample plots of poplar plantation forests in the western sandy area of Liaoning, covering a total area of approximately 24,000 hectares. These plots are categorized into age groups based on national forestry standards^26^. To estimate the total soil carbon stock of poplar plantation forests in this region, we use the following formula:

4 $$C={\sum }_{i=0}^{n}\left({S}_{Ci}{S}_{i}\right)$$

In the formula, *C* represents the soil carbon stock (t/ha) for the entire area; *S*_*ci*_ is the soil carbon stock (t/ha) for the *i*-th plot; and *S*_*i*_ is the area of the *i*-th plot (ha).

#### Data analysis

Data organization and tabulation were performed using Excel. One-way analysis of variance (ANOVA) was conducted to assess the significance of differences using IBM SPSS Statistics 26 software. Bivariate Pearson correlation analyses were carried out to examine the relationships between stand density, diameter at DBH growth, TH growth, soil moisture content, and soil carbon content in the poplar plantations of the western Liaoning sandy area. Correlation plots were generated using Origin 2021, and the plots display means and standard deviations. The soil carbon stock of poplar plantation forests in the western Liaoning wind and sand area was estimated and visualized using ArcMap 10.2.

## Results

### Impact of different age groups and stand density on diameter at DBH and TH growth in poplar plantations

**Figure. 2:**
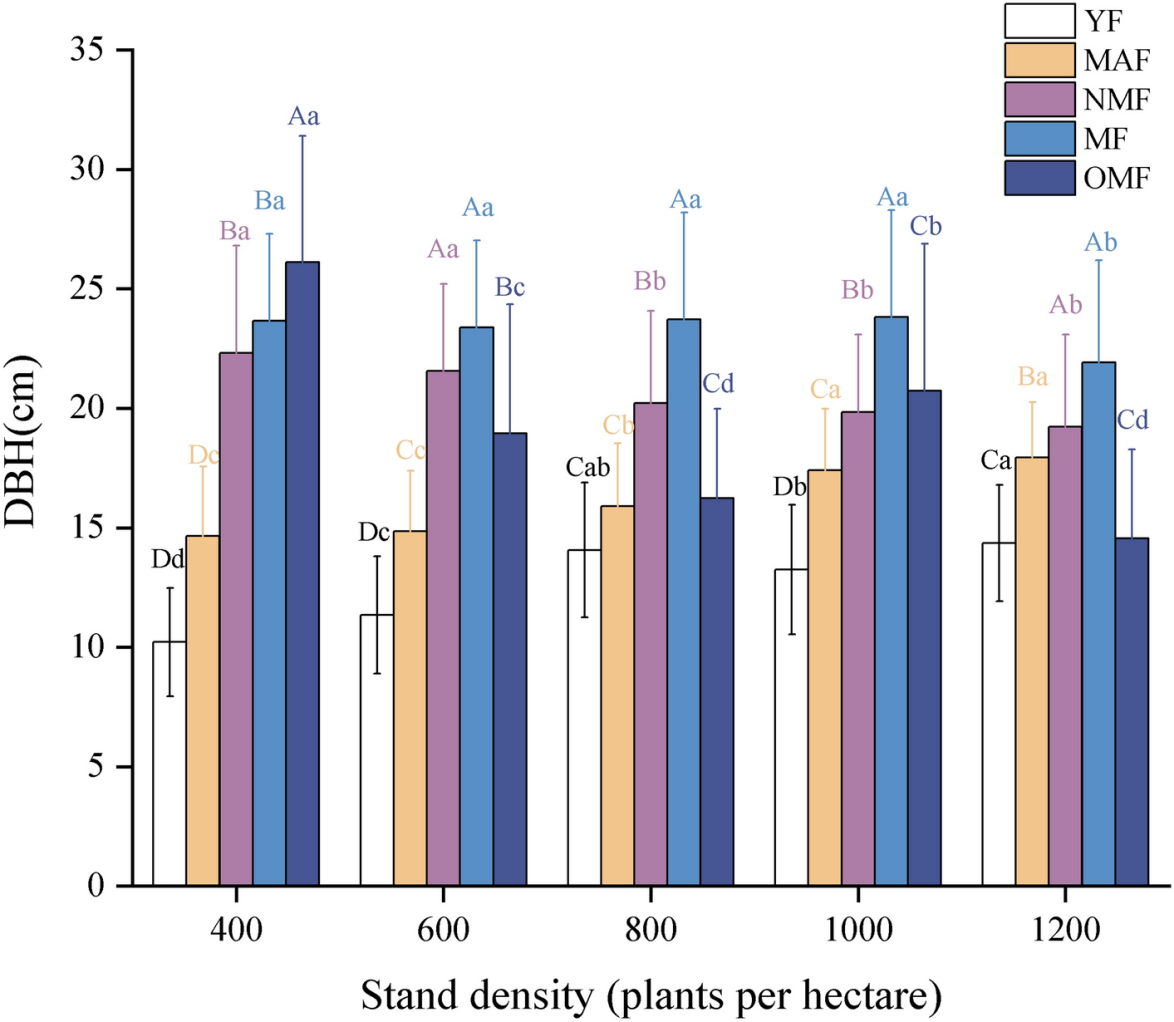
DBH of *Poplar* plantation. YF, MAF, NMF, MF, OMF denote young forest, middle aged forest, near mature forest, mature forest, over mature forest. different capital letters denote significant differences between age group, and different lowercase letters denote significant differences between stand densities (*p* < 0.05).

As illustrated in Fig. 2, there is a notable variation in the diameter at DBH growth of poplar plantation forests across different growth stages. DBH growth generally increases with age. Specifically, in stands with a density of 400 trees per hectare, the average DBH of young forests was the smallest at 10.2 cm, while the DBH of overmature forests was the largest at 26.1 cm, representing an average increase of 14.9 cm.

In young and middle-aged forests, DBH also increased significantly (p < 0.05) with higher stand density, suggesting better DBH growth at these stages. On the contrary, DBH decreased (p < 0.05) with higher stand density in near-mature forests. At the mature forest stage, DBH showed relative stability with no significant trend as stand density increased, but at the over-mature forest stage stand density increased and DBH decreased.

**Figure. 3:**
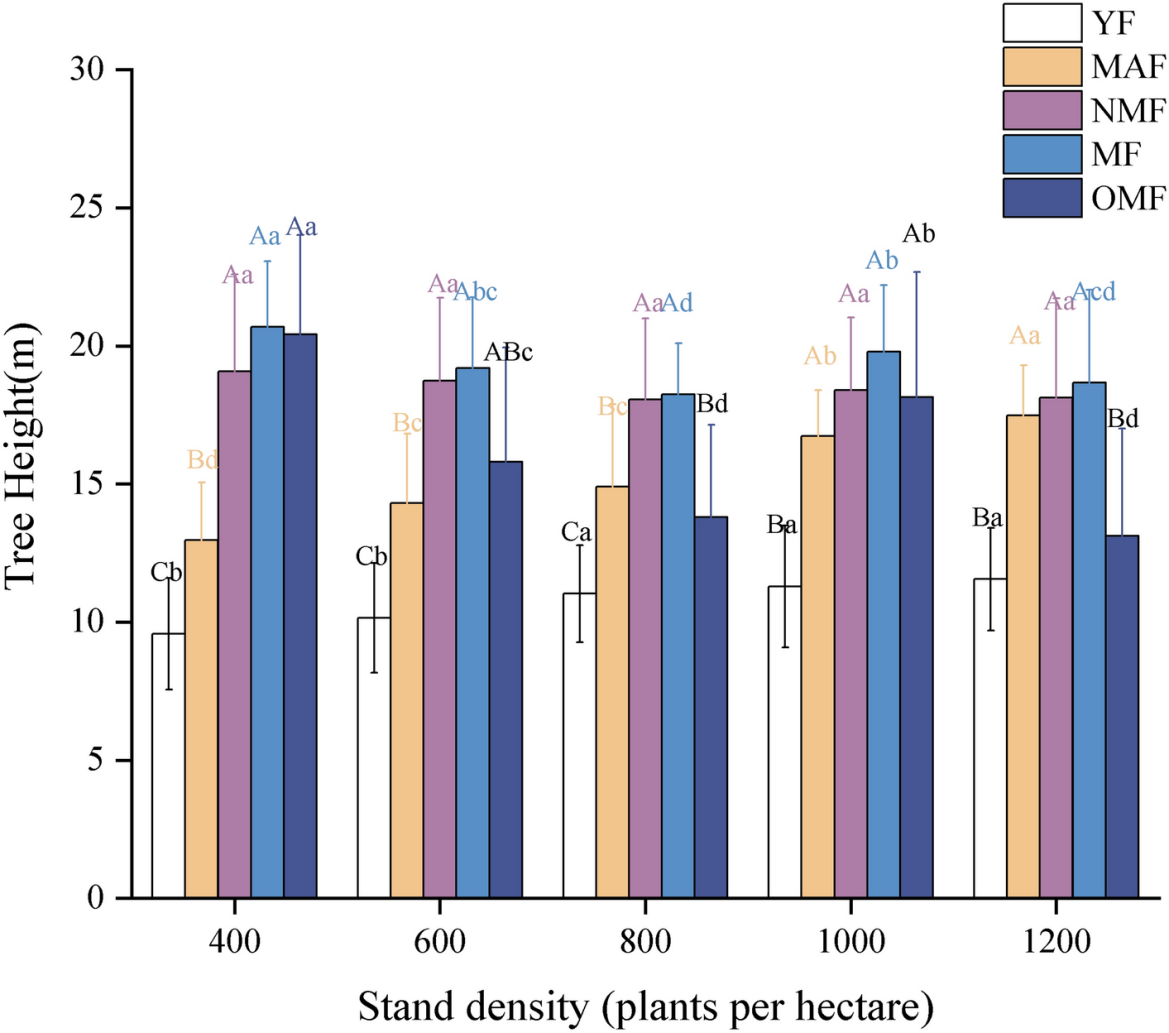
Height of *Poplar* plantation. YF, MAF, NMF, MF, OMF denote young forest, middle aged forest, near mature forest, mature forest, over mature forest. different capital letters denote significant differences between age group, and different lowercase letters denote significant differences between stand densities (*p* < 0.05).

As shown in Fig. 3, the growth in TH of poplar plantation forests closely follows the pattern observed for diameter at DBH. TH growth generally increases with stand age, with young forests exhibiting the lowest average height of 10.72 m, and mature forests achieving the highest average height of 19.32 m.

For young and middle-aged forests, TH increased significantly with higher stand density (*p* < *0.05*). However, in near-mature and overmature forests, TH decreased significantly with increasing stand density (*p* < *0.05*). This indicates that while higher stand densities promote better TH growth in younger forests, they negatively impact TH growth in near-mature, mature, and overmature forests.

This study reveals that the impact of different stand densities on poplar plantation forests in the wind-sand area of western Liaoning diminishes with increasing stand age. The effects are more pronounced in young, middle-aged, and near-mature forests, while the influence on mature and overmature forests is less significant. As stand density increases, the growth conditions for young and middle-aged forests continue to improve. However, for near-mature, mature, and overmature forests, growth becomes increasingly constrained with higher densities. Additionally, while stand diameter at DBH and TH generally exhibit a stable upward trend from young to mature forests, there may be a decline in growth in overmature forests.

### Impact of age groups and stand density on soil moisture content in poplar plantations

**Figure. 4:**
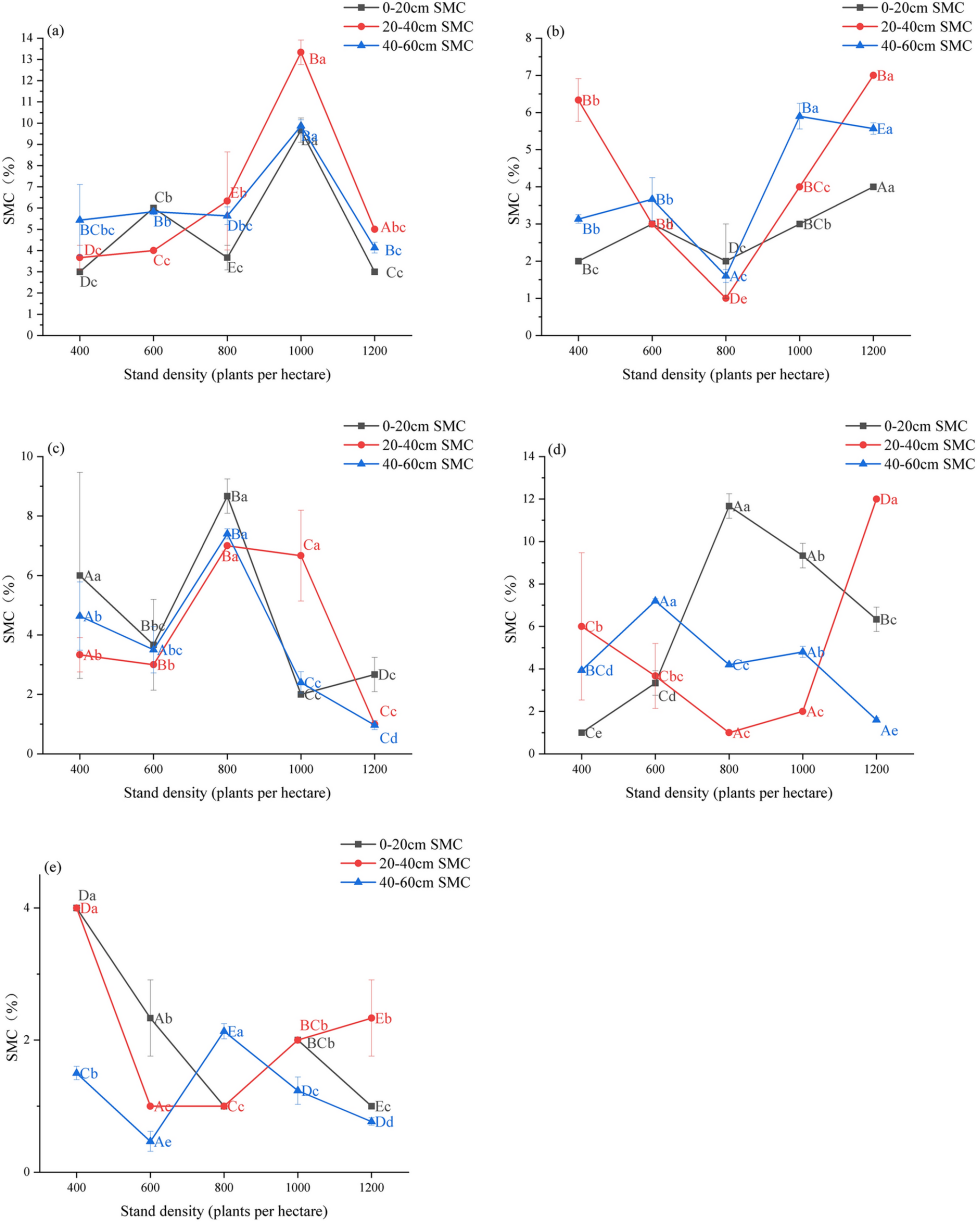
SMC of different age groups of *poplar* plantation under different stand density. (**a**–**e**) denote YF, MAF, NMF, MF, OMF. different capital letters denote significant differences between stand densities, and different lowercase letters denote significant differences between soil layers (*p* < 0.05).

As stand density increases, soil moisture content in young forests shows a general upward trend (*p* < *0.05*, Fig. 4a). For middle-aged forests, soil moisture content initially decreases and then increases (Fig. 4b). In near-mature forests, soil moisture content exhibits a trend of increasing followed by a decrease (*p* < *0.01*, Fig. 4c). There is no significant trend observed in the mature forest stage (*p* < *0.05*, Fig. 4d). In overmature forests, soil moisture content consistently decreases with increasing stand density, and overall, it remains lower compared to other age stages (*p* < *0.05*, Fig. 4e). Specifically, the average soil moisture content at a stand density of 1000 trees per hectare is highest in young forests at 11%, while it is lowest in overmature forests at 1.3%.

### Impact of age groups and stand density on soil carbon content in poplar plantations

**Figure. 5:**
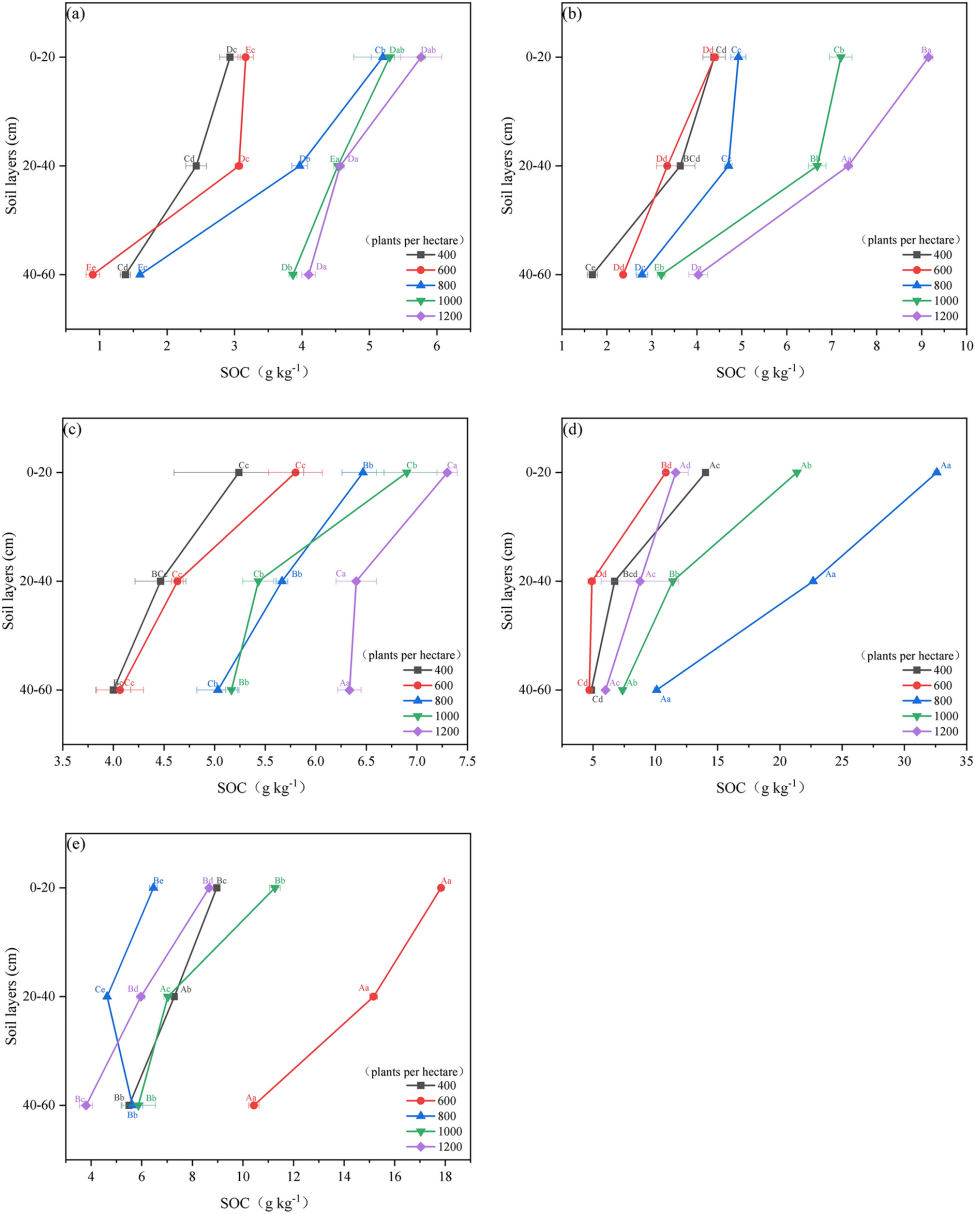
SOC of different age groups of *poplar* plantation under different stand density. (**a**–**e**) denote YF, MAF, NMF, MF, OMF. different capital letters denote significant differences between stand densities, and different lowercase letters denote significant differences between soil layers (*p* < 0.05).

In the 0–60 cm soil layer, soil carbon content in poplar plantation forests generally increases with higher stand density in the juvenile, middle-aged, and near-mature stages, peaking at 1200 trees per hectare and reaching a minimum at 400 trees per hectare (*p* < *0.05*, Fig. 5a–c). In the mature forest stage, soil carbon content initially increases and then decreases with increasing stand density, peaking at 800 trees per hectare and reaching a minimum at 1000 trees per hectare (*p* < *0.05*, Fig. 5d). For overmature forests, soil carbon content shows minimal variation with stand density, peaking at 1000 trees per hectare and being lowest at 800 trees per hectare (*p* < *0.05*, Fig. 5e). The average soil carbon content is highest at 800 trees per hectare in mature forests (21.79 g kg^-1^) and lowest at 400 trees per hectare in young forests (2.25 g kg^-1^). Across all stand densities, soil carbon content decreases with soil depth, being highest at 0–20 cm and lowest at 40–60 cm depth.

### Correlation analysis of stand growth, density, soil moisture, and soil carbon content in poplar plantations across age groups

Correlation analysis in this study assessed the relationships between stand density, diameter at DBH, TH, soil moisture content, and soil carbon content across different age groups of poplar plantations in the sandy area of western Liaoning. The results from bivariate Pearson correlation tests revealed that the impact of stand density on various metrics decreased with increasing forest age.

**Figure. 6:**
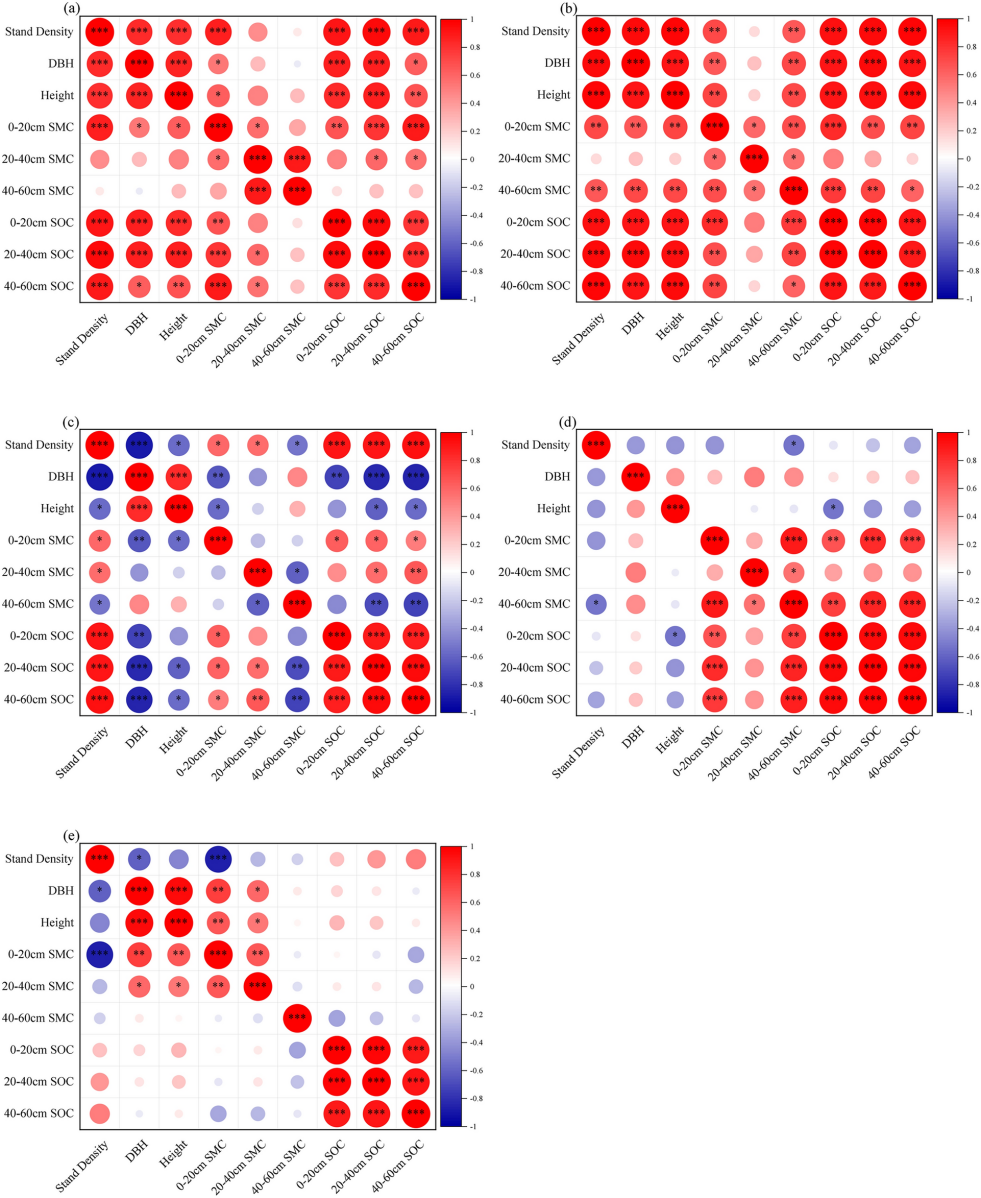
Correlation analysis of stand density, DBH, TH, Soil moisture, SOC in different age groups of *Poplar* plantation. (**a**–**e**) denote YF, MAF, NMF, MF, OMF. Asterisks show the significance of correlation analysis of each index (*** p < 0.001, ** p < 0.01, * *p* < 0.05).

In young forests, significant positive correlations were found between stand density and growth metrics (DBH and TH) as well as soil carbon content (*p* < *0.01*, Fig. 6a). As stand density increased, both growth rates and soil carbon content improved. In middle-aged forests, similar positive correlations persisted between stand density, DBH growth, TH growth, and soil carbon content (*p* < *0.01*, Fig. 6b).

In near-mature forests, there was a significant positive correlation between stand density and soil carbon content (*p* < *0.001*, Fig. 6c), but growth metrics such as DBH and TH showed mixed results. Stand density positively influenced DBH growth and TH (*p* < *0.001*, Fig. 6c), but negatively affected the relationship between these metrics and soil carbon content (*p* < *0.05*, Fig. 6c). In mature forests, stand density was positively correlated with soil moisture content (*p* < *0.01*, Fig. 6d), while other metrics showed no significant correlations.

In over-mature forests, stand density was negatively correlated with DBH growth, TH growth, and soil moisture content (*p* < *0.05*, Fig. 6e), but DBH growth remained positively correlated with TH growth (*p* < *0.01*, Fig. 6e) and soil moisture content (*p* < *0.05*, Fig. 6e). Additionally, the correlation between soil moisture content and other indicators decreased with increasing soil depth.

### Estimation of soil carbon stocks in poplar plantations in the Liao western sandy area

**Figure. 7:**
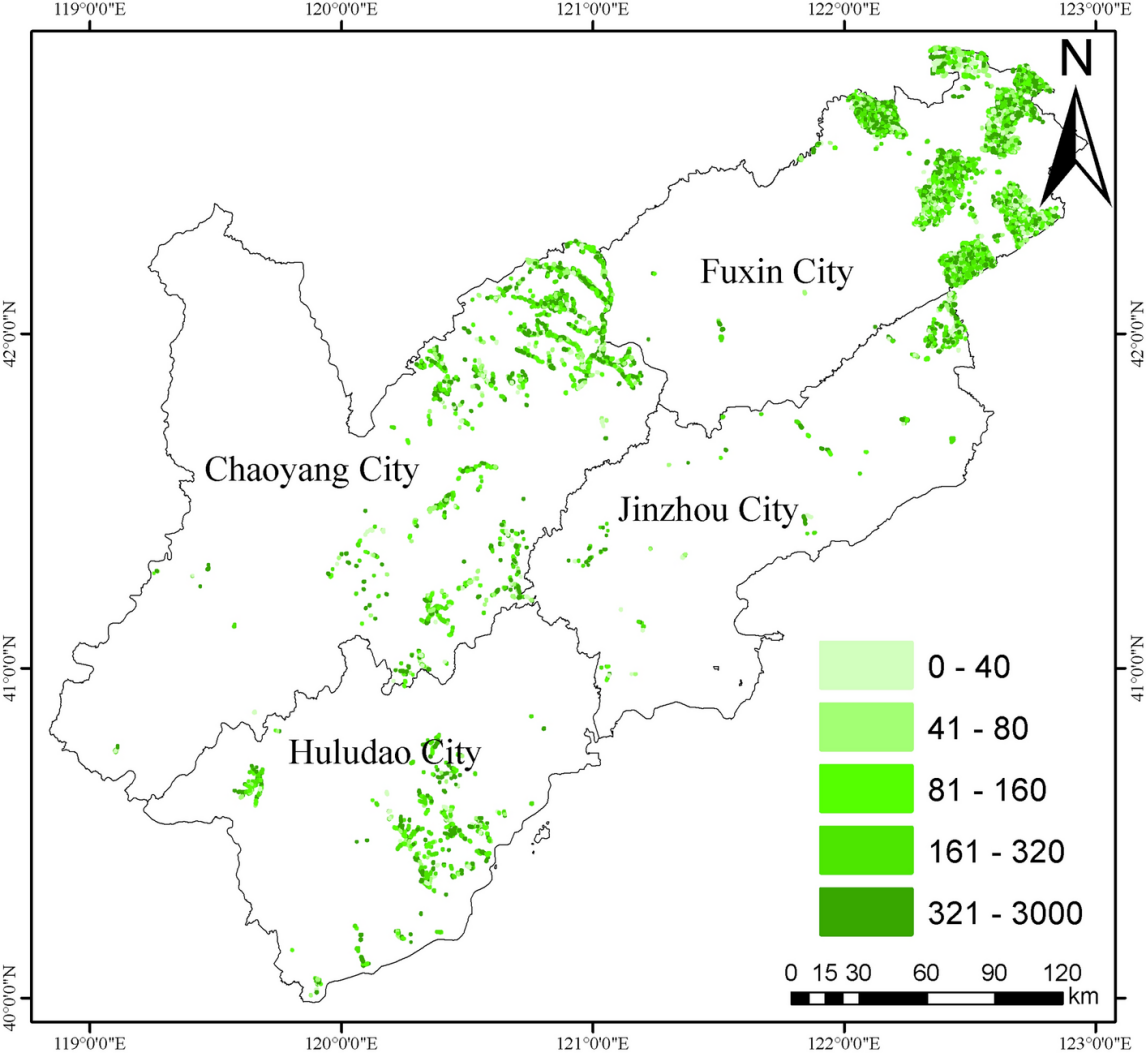
Distribution pattern of soil carbon stocks of *poplar* plantation in sandy area of western Liaoning province. Green from shallow to deep denote the soil carbon stocks values range from low to high.

In this study, the soil carbon stock of poplar plantation forests in the west of Liaoning sandy area was estimated to be approximately 2,989,693.44 tons. The highest soil carbon stock was found in Fuxin City at 2,011,691.21 tons, followed by Chaoyang City at 650,158.44 tons. Huludao City had a lower stock of 202,359.57 tons, and Jinzhou City had the lowest at 125,483.76 tons (Table 2). The distribution pattern shows that the highest soil carbon stocks are concentrated in the northeastern part of Fuxin City, followed by Chaoyang City and the south-central part of Huludao City. The lowest stocks are in the northeastern region of Jinzhou City (Fig. 7).

**Table 2:** Estimation of soil carbon stocks in semi-arid aeolian sand area of western Liaoning province.

	Age group	Area/(ha)	Soil type	Soil carbon stocks/(t)
Fuxin City	YF	10,416.8	Sandy land	894,698.8
	MAF	3320	Sandy land	335,153.5
	NMF	1412.9	Sandy land	177,457.1
	MF	1973.1	Sandy land	324,055.3
	OMF	1715.6	Sandy land	280,326.8
Chaoyang City	YF	1775.6	Sandy land	135,717.6
	MAF	1701	Sandy land	177,229.9
	NMF	502.5	Sandy land	67,261.06
	MF	742.8	Sandy land	110,710.3
	OMF	986.1	Sandy land	159,239.6
Huludao City	YF	1210.4	Sandy land	125,853.8
	MAF	359.7	Sandy land	39,649.99
	NMF	163.5	Sandy land	19,504.37
	MF	104.7	Sandy land	7795.39
	OMF	58.4	Sandy land	9556.005
Jinzhou City	YF	508.9	Sandy land	37,556.27
	MAF	184.2	Sandy land	19,302.37
	NMF	215.5	Sandy land	26,411.53
	MF	211.6	Sandy land	26,641.78
	OMF	92.6	Sandy land	15,571.81

## Discussion

### Effects of stand density and age group on the diameter and height of poplar plantations

Stand density is a crucial factor influencing forest growth and ecological functions^27^. As observed in this study, diameter at DBH and TH generally increase with forest age. This is consistent with Lan et al.^28^, who noted that the number of trees in a stand decreases as it ages. Effective forest management practices, including adjusting stand density, can significantly impact tree growth^29,30^.

Primicia et al.^31^argued that in the early stages, when competition between trees is less intense, higher stand densities can enhance growth by providing more vertical space and nutrients. This perspective is supported by the present study, where DBH and TH showed a strong positive correlation with stand density during the young and intermediate stages. This finding is in line with previous studies on oil pine^32^, cypress^33^, and other plantation forests^34^. This supports Hypothesis 1, which posits that increasing stand density enhances DBH and TH in young and intermediate poplar plantations.

However, at the near-mature, mature, and over-mature stages, increased stand density was associated with a decrease in DBH and TH. This trend aligns with the findings of Pachas et al.^35^on silver acacia. As trees age, competition for resources intensifies^36^. Excessive stand density can exacerbate this competition, limiting individual tree growth due to restricted access to nutrients and growing space.

In this study, the average DBH and TH increased rapidly with stand age but showed a marked slowdown after reaching a certain stage, with a decreasing trend evident in mature and over-mature forests. This pattern is consistent with previous research^37–39^ and supports Hypothesis 2, which states that DBH and TH increase with age but may slow down after maturity.

This suggests that while stand density positively influences growth during the young and intermediate stages, it becomes less effective, or even detrimental, as the forest matures. Overly dense stands can lead to negative outcomes, including increased competition for resources and reduced overall stand health. Therefore, it is crucial to implement adaptive density management strategies that account for the different growth stages of the forest.

Implications for Forest Management:

- *Early stages*: Higher stand densities can enhance DBH and TH, promoting growth and productivity.
- *Mature stages*: Density adjustments are necessary to prevent excessive competition, which can hinder growth and affect stand health. Proper management can maintain productivity and improve economic benefits.

In summary, effective stand density management is essential for optimizing tree growth and forest productivity. As forests transition through various age stages, adjusting density according to the specific needs and characteristics of each stage can help achieve optimal growth benefits and ensure long-term forest health.

### Effects of stand density and age group on soil moisture in Poplar plantations

Soil moisture plays a crucial role in tree growth, particularly in arid and semi-arid regions where it can be a limiting factor^40,41^. The response of soil moisture to changes in forest cover is vital for understanding how forest management can affect water retention^42^.

In young and middle-aged forests, and at the near-mature stage, a positive correlation between soil moisture content and stand density was observed. This finding aligns with Liu et al.^43^, who noted that higher stand densities reduce soil exposure to sunlight and evaporation, thereby retaining more moisture. Additionally, the increased tree cover helps to protect the soil from direct sunlight and reduce evaporation, while the root systems contribute to better water retention^44^.

Conversely, in mature and over-mature forests, the correlation between soil moisture content and stand density becomes negative. This is consistent with Cui et al.^45^ and Xiong et al.^46^, who found that higher stand densities in older forests can lead to increased competition for water among tree roots, reducing soil moisture. Mature trees consume more water, leaving less available moisture in the soil. The decreased correlation between soil moisture and various indicators with increasing soil depth further suggests that surface soil moisture is more affected by stand density changes in wind-sand areas.

Understanding these dynamics is crucial for optimizing forest management strategies, especially in arid and semi-arid regions. Managing stand density appropriately can enhance soil moisture retention in younger forests and mitigate negative impacts in mature stands. By considering these interactions, forest managers can promote sustainable land development and improve ecosystem services^47,48^.

### Effects of stand density and age group on soil carbon content in Poplar plantations

The scientific management of plantation forests in arid zones can enhance soil organic carbon and improve soil quality, especially in ecologically fragile areas^49,50^. This study supports the view that soil carbon content is positively correlated with stand age^51–53^, which aligns with our findings that soil carbon content in poplar plantations increases with stand age. This increase is attributed to the expanding root systems and the stabilization of soil carbon through improved ecosystem complexity and stability^47,48,54^.

Under similar age groups, higher stand densities were associated with higher soil carbon content, consistent with Gebremeskel et al.^55^ and Na^56^. Increased vegetation cover and root mass at higher densities contribute to greater soil organic matter input and better conditions for carbon stabilization^57^. High-density plantings reduce soil exposure to sunlight, minimize evaporation, and enhance soil protection from wind and water erosion^58^. These factors collectively improve soil structure, increase porosity, and boost microbial activity, all of which facilitate soil carbon accumulation^59,60^.

The study also found a gradual decrease in soil carbon content with increasing soil depth, which aligns with findings by Cao et al.^61^ and Wang et al.^62^, but differs from some studies^63,64^. The higher carbon content in shallow soils is due to greater organic matter input and biological activity at the surface, while deeper soils receive less organic matter and exhibit less biological activity^65,66^. This results in a lower carbon content in deeper soil layers.

Overall, effective forest management should consider the impacts of stand age and density on soil carbon content. Strategies to optimize age structure, increase organic matter inputs, and protect soil surfaces can enhance soil carbon storage and promote ecosystem stability^67–70^.

## Conclusion

In the west of Liaoning sandy area, poplar plantation forests exhibit increasing diameter at DBH, TH, and soil carbon content with stand age. During the young and middle-aged stages, higher stand density positively impacts TH, DBH, soil moisture, and soil carbon content. However, in near-mature, mature, and over-mature stages, increased stand density results in reduced TH, DBH growth, and soil moisture, though soil carbon content continues to rise. The total soil carbon stock was approximately 3.0 × 10^6^ t, highest in Fuxin City and lowest in Jinzhou City. It is recommended to maintain stand densities of about 1200 ha^−1^ for young to middle-aged forests and 800 ha^-1^ for mature forests. This guidance is vital for the effective afforestation and management of poplar plantations in the region.

## Supplementary Information

Supplementary Information.

## Data Availability

All data generated or analysed during this study are included in this published article and its supplementary information files.
